# Effect of Post-Harvest Management on *Aspergillus flavus* Growth and Aflatoxin Contamination of Stored Hazelnuts

**DOI:** 10.3390/toxins18010038

**Published:** 2026-01-11

**Authors:** Alessia Casu, Giorgio Chiusa, Eugenio Zagottis, Giuseppe Genova, Paola Battilani

**Affiliations:** 1 Department of Sustainable Crop Production, Università Cattolica del Sacro Cuore, Via Emilia Parmense 84, 29122 Piacenza, Italy; alessia.casu@unicatt.it (A.C.); giorgio.chiusa@unicatt.it (G.C.); 2 Soremartec Italia S.r.l., Piazzale Pietro Ferrero 1, 12051 Alba, Italy; eugenio.zagottis@ferrero.com (E.Z.); giuseppe.genova@ferrero.com (G.G.)

**Keywords:** aflatoxins, *Aspergillus flavus*, drying, food safety, hazelnut, mycotoxins, post-harvest management, storage, Azerbaijan

## Abstract

Hazelnut (*Corylus avellana* L.) is a major crop in the Caucasus region, but its safety is often threatened by *Aspergillus flavus* colonization and aflatoxin contamination. Although aflatoxins (AFs) are strictly regulated in the EU, the influence of post-harvest practices on fungal persistence and AF accumulation remains poorly defined. A three-year study was conducted to evaluate the effects of drying protocols, storage temperature, and conservation practices on fungal growth and AF occurrence in hazelnuts from three producing regions of Azerbaijan. Freshly harvested nuts were subjected to two drying regimes: good drying (sun-exposed, mixed, protected from rewetting) and bad drying (shaded, piled, rewetted). After drying, samples were stored at cold (8–10 °C) or room temperature (18–22 °C). Fungal prevalence was determined by CFU counts with morphological and qPCR identification of *Aspergillus* section *Flavi*. AFs were quantified by HPLC, and water activity (a_w_) was monitored during storage. Drying emerged as the decisive factor: bad drying consistently resulted in markedly higher fungal loads for *A.* section *Flavi*, with mean counts up to 1.3 log10 (CFU/g), compared with 0.8 log10 (CFU/g) under good drying, representing a 7-fold increase. In contrast, storage temperature and shell condition had negligible effects when nuts were properly dried. Aflatoxins were consistently below the 5 µg/kg EU limit for AFB_1_ in traced and well-dried samples, whereas market samples occasionally exhibited AFB_1_ concentrations >450 µg/kg. These findings highlight drying efficiency as the key determinant of fungal persistence and AF risk in hazelnut post-harvest management.

## 1. Introduction

Hazelnut (*Corylus avellana* L.) is a crop of major economic relevance, appreciated worldwide for its nutritional value and wide use in the food industry. Hazelnuts are rich in unsaturated fatty acids, proteins, vitamins, and bioactive compounds, making them valuable for direct consumption and as key ingredients in confectionery, bakery, and chocolate products [1]. Azerbaijan is among the leading global producers, ranking fourth in 2023, and hazelnut cultivation plays a crucial role in both domestic supply and international trade [2,3].

Despite their economic importance, hazelnuts are susceptible to quality deterioration and microbial contamination in the production chain [4,5]. Post-harvest stages are particularly critical, since handling practices may strongly affect nut quality and safety. Factors such as inadequate drying, delayed processing, and suboptimal storage conditions can compromise kernel integrity, shorten shelf life, and promote fungal colonization [6,7]. Among the fungal species associated with hazelnuts, those belonging to *Aspergillus* section *Flavi* are of particular concern because of their ability to produce aflatoxins (AFs), secondary metabolites with high toxicological relevance. Within this section, *A. flavus* is the species most frequently isolated from hazelnut orchards, soils, and defective kernels in major producing regions, and is recognized as the primary etiological agent responsible for aflatoxin B_1_ contamination in hazelnuts [7,8,9]. Aflatoxin B_1_ (AFB_1_), the most potent compound of this group, is classified as a Group 1 carcinogen by the International Agency for Research on Cancer [10].

Regulatory authorities worldwide have established strict maximum limits for AFs in food products. In the European Union, Commission Regulation (EU) No. 2023/915 sets the legal thresholds for hazelnuts intended for direct consumption at 5 µg/kg for AFB_1_ and 10 µg/kg for total AFs, with slightly higher limits for nuts intended for sorting or further processing. Exceeding these thresholds not only poses severe risks for consumer health but also results in product rejection, economic losses, and significant trade restrictions [9,10,11,12]. The European Commission Rapid Alert System for Food and Feed (RASFF) has repeatedly issued notifications regarding AF contamination in hazelnuts, with Azerbaijan among the countries most frequently involved in recent years [13,14].

AF contamination in hazelnuts can occur at different stages of the value chain, including the growing period in the orchard, during harvest, and in post-harvest processing. However, several studies have highlighted the post-harvest phase as particularly decisive, since improper drying and storage conditions provide favorable environments for *Aspergillus flavus* growth and AF accumulation [7,15,16]. Traditional sun-drying on the ground or under variable weather conditions has been associated with elevated risks, whereas mechanical drying methods and well-controlled storage can significantly reduce contamination. Similarly, inadequate storage facilities, especially those with high relative humidity and poor aeration, have been recognized as major contributors to AF occurrence [1,17].

To counteract deterioration, drying is a prerequisite before storage, as high moisture content accelerates fungal growth and lipid oxidation. Storage under controlled conditions, with low temperature and relative humidity below 60%, has been shown to slow enzymatic and chemical oxidation processes and to limit fungal development, including AFs-producing species [17,18,19]. Moreover, modified or inert atmospheres, created through nitrogen or carbon dioxide saturation, can further contribute to preserving quality by reducing oxygen availability and slowing oxidative reactions [20,21]. Despite these strategies, scientific data concerning hazelnut storage techniques remain relatively scarce, and little information is available for traditional production systems such as those applied in Azerbaijan.

In Azerbaijan, hazelnut post-harvest practices are still largely based on smallholder production systems, with limited mechanization and reliance on traditional methods. Hazelnuts are commonly harvested by shaking the branches to make the nuts fall to the ground, followed by mechanical dehusking using decorticating machines. In some cases, early harvesting is performed directly from the tree; in such instances, nuts with green husks are usually sun-dried in layers to facilitate detachment, which is then completed mechanically. The in-shell nuts are subsequently transported to processing facilities for cleaning and drying. Storage can be under conditions in which temperature and humidity are difficult to control [2,20]. These factors, combined with the country’s climatic variability, increase the vulnerability of hazelnuts to fungal infection and AF production, undermining product quality and marketability [22].

The aim of this study was therefore to investigate the effects of key post-harvest variables, including drying methods, storage temperature, and conservation practices, on *A. flavus* growth and AF contamination in Azerbaijani hazelnuts. By monitoring nuts across different handling, storage conditions and time periods, this work aimed to provide insights into the factors most critical for *A. flavus* activity and AF contamination during the post-harvest phase.

## 2. Results

### 2.1. Fungal Growth in Selected Orchards

In a preliminary ANOVA, each factor was evaluated individually to identify which variables significantly influenced fungal contamination before building the full multifactorial model. This screening analysis showed that orchard of origin and year had a significant effect on *A*. section *Flavi* growth (*p* < 0.01), and drying method also strongly influenced contamination levels. In contrast, storage phase and storage temperature did not significantly affect *A*. section *Flavi* when analyzed as singularly.

Based on these results, only the factors that significantly impacted *A*. section *Flavi* (orchard, year, drying method) were included in the subsequent multifactorial ANOVA, where their combined effects and interactions were evaluated.

**Figure 1:**
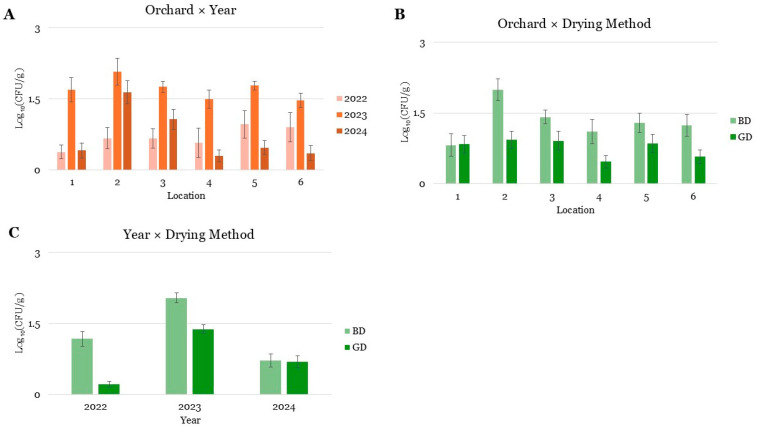
Interaction between Orchard x Year (**A**), Orchard x Drying Method (**B**) and Year x Drying Method (**C**) on *A.* section *Flavi* growth (CFU/g) in hazelnut samples collected from selected orchards in Azerbaijan. Bars represent mean values ± standard errors derived from multiple independent samples within each factor combination.

Overall, production year was the most influential factor (*p* < 0.01 for all fungal groups), with the 2023 harvest season showing substantially higher fungal loads compared to 2022 and 2024. The drying method also had a strong impact: across most fungal groups, BD resulted in significantly higher CFU/g compared to GD, underscoring the importance of proper post-harvest handling. For *A.* section *Flavi*, growth peaked in 2023, with mean counts approximately two times higher than in 2022 and 2024 (Table 1). BD strongly favored fungal growth compared to GD (*p* < 0.01). Moreover, certain orchards (e.g., orchard #2) consistently displayed higher growth levels compared to others (*p* < 0.01). Significant O × Y, O × D and Y × D interactions indicate that the impact of drying practices varied between years and across locations, suggesting that BD was particularly detrimental under specific environmental conditions (Figure 1).

### 2.2. Fungal Growth in Market Samples

For market samples, the one-factor ANOVA was also used as preliminary step. This screening analysis showed that market source and year were the only factors significantly influencing *A*. section *Flavi* growth (*p* < 0.01), while phase, drying practices reported by farmers, storage in shell, and storage temperature did not exert significant effects when evaluated individually.

Based on these preliminary results, only market source and year, the significant drivers for *A*. section *Flavi*, were included in the subsequent multifactorial ANOVA to explore their combined effects and interactions.

Across all fungal genera, 2023 exhibited the highest growth levels, confirming that climatic conditions during this production season strongly favored fungal proliferation. Significant differences among market sources were also detected, particularly for *Aspergillus* spp. and *Penicillium* spp. (*p* < 0.01). For *A*. section *Flavi*, growth peaked in 2023, that showed fungal counts significantly higher than in 2022 and 2024 (Table 2).

Market sources (M) #1, #3, #4, and #6 showed consistently higher growth levels compared to other sources. The interaction M × Y was significant, indicating that some areas were particularly vulnerable during the 2023 season.

### 2.3. Confirmation of A. flavus and A. parasiticus Identification

The 25 monosporic isolates belonging to *A.* section *Flavi* displayed highly homogeneous colony morphologies when grown on 5/2 agar. Most isolates developed the characteristic ivy-green mycelium with granular to powdery texture and pale yellow to orange reverse coloration, consistent with descriptions of *A. flavus*, whereas a single isolate exhibited a cress-green color and more velvety surface, typical of *A. parasiticus* [23].

**Figure 2:**
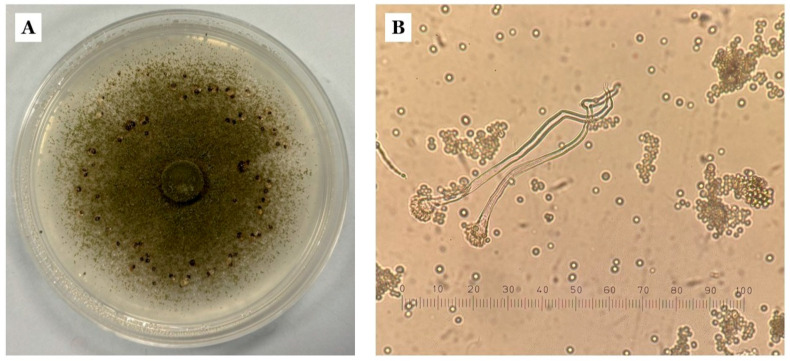
Macroscopic and microscopic features of *Aspergillus* section *Flavi* from 5/2 agar. (**A**) Colony morphology of *A. flavus* grown on 5/2 agar for 5–7 days at 31 °C, showing the characteristic olive-green conidial layer and the presence of sclerotia. (**B**) Optical microscopy image (40× magnification) displaying the typical unbranched conidiophores ending in globose vesicles and rough-walled conidia.

Under stereomicroscope, *A. flavus*-like isolates showed densely conidial surfaces and frequent sclerotial structures, while the *A. parasiticus*-like isolate lacked visible sclerotia. Simple slide preparations confirmed the presence of typical *Aspergillus*-type conidiophores and rough-walled conidia, supporting the genus-level identification (Figure 2).

Molecular identification using SYBR Green real-time PCR confirmed the morphological observations for most isolates: 20 were positively identified as *A. flavus*, while one isolate was confirmed as *A. parasiticus*, consistent with its atypical colony color. Interestingly, four isolates failed to amplify with both *A. flavus* and *A. parasiticus* primer sets, presumably because they belong to other species within *A.* section *Flavi*.

### 2.4. Aflatoxin Contamination

The quantification of AFB_1_ and total AFs revealed a clear difference between samples from selected orchards and those from the local market.

For the selected orchards, contamination levels were consistently low. Across all samples and years, AFB_1_ concentrations remained below 1.5 µg/kg and total AFs did not exceed 2.5 µg/kg, well below the maximum limits set by the European Commission for unprocessed hazelnuts.

In contrast, market samples showed high variability and, in some cases, severe contamination. While some were within legal limits, others reached critically high levels, with AFB_1_ concentrations exceeding 450 µg/kg and total AFs surpassing 500 µg/kg. These extreme cases were predominantly associated with in-shell samples, combined with BD practices and storage at room temperature (Table 3).

Moreover, in three market samples (#6, #7 and #8 from 2022/2023 production season), G-type AFs, mainly produced by *A. parasiticus*, were identified.

Interestingly, all samples with elevated AF levels belonged to the PH3 stage, suggesting that prolonged storage durations, together with suboptimal drying and variable storage conditions, are key drivers of AF accumulation.

### 2.5. Water Activity

The water activity (a_w_) of hazelnut samples was monitored monthly from October to June under all the different post-harvest conditions, including drying method, storage temperature and storage in-shell or shelled.

Overall, a_w_ values remained stable throughout the monitoring period, generally ranging between 0.61 and 0.67 for selected orchard samples and between 0.60 and 0.79 for market samples (Supplementary Table S1). No clear seasonal patterns or abrupt fluctuations were observed, indicating that both drying practices and storage conditions allowed hazelnuts to maintain a relatively constant moisture equilibrium.

When comparing the different post-harvest variables, only minor and inconsistent variations were detected. Considering the drying method, no systematic differences were observed, as a_w_ trends were almost identical across paired samples subjected to good or bad drying.

Regarding storage temperature, samples stored at room temperature occasionally showed slightly higher a_w_ values towards the end of storage, but the differences were marginal (≤0.02–0.03) and not consistent across samples.

For storage in-shell or shelled, the presence of the shell did not appear to significantly influence a_w_ dynamics, with values comparable to those of shelled samples.

Importantly, across all treatments and sampling times, a_w_ values stabilized below 0.70, a threshold generally considered restrictive to produce toxins by mycotoxigenic fungi such as *Aspergillus* spp.

## 3. Discussion

The results from this study confirm that drying is the most critical step in the post-harvest management of hazelnuts, with BD conditions consistently leading to higher fungal loads across both orchard and market samples. Improper or delayed drying creates favorable conditions for fungal proliferation, even when a_w_ remains below 0.70, threshold generally considered restrictive for mycotoxigenic fungi.

Experimental investigations on artificially inoculated hazelnuts have demonstrated that suboptimal drying temperatures (30–40 °C) prolonged processing time and promoted *A. flavus* growth, while more efficient drying at 45 °C prevented both fungal development and AF formation [1]. Likewise, a survey conducted on ready-to-eat hazelnuts from multiple cultivars and production areas has showed that drying, although effective in reducing fungal loads, does not fully eliminate stress-resistant genera such as *Aspergillus* and *Penicillium* [24].

Importantly, this study directly compared two realistic sun-drying practices widely used by smallholders in Azerbaijan, defined as GD and BD, rather than testing controlled mechanical drying. Unlike experimental settings employing mechanical or forced-air dryers [25,26,27], it was evaluated post-harvest behaviour under field-relevant, low-technology conditions that reflect the constraints of the Azerbaijani sector. This distinction is essential: while the literature consistently shows that mechanical drying at 40–50 °C reduces drying time, limits fungi growth, and improves storage stability, these systems are still economically and logistically inaccessible for most small producers. These findings therefore provide evidence-based guidance for improving traditional sun-drying itself, demonstrating that even within low-input systems, better practices (thin layers, turning, protection from rewetting) substantially reduce fungal risk compared to suboptimal BD practices.

In Azerbaijan, BD practices remain widespread, often involving shaded areas, piling without turning, or exposure to rewetting. These traditional methods, dictated by limited infrastructure, explain the recurrent AF contamination observed in certain production areas. Similar challenges have been reported for other major hazelnut-producing regions, where prolonged sun-drying under humid or rainy conditions (as typical in the Black Sea area) leads to extended drying periods of 15–30 days and increased susceptibility to fungal growth and AF formation [26,28]. These observations therefore align with international evidence highlighting that slow drying, rewetting, and thick layers are key drivers of contamination.

Given the increasing economic importance of hazelnut production, such deficiencies pose a major challenge. Persistent contamination risks undermining international market access, as reflected by recent RASFF notifications that have limited Azerbaijani exports to the EU, resulting in substantial economic losses. Moreover, if noncompliant lots are redirected toward less regulated markets, they may still enter the global food chain, creating broader food safety concerns [2,13].

The fungal community observed in this study was dominated by *A.* section *Flavi* and *A.* section *Nigri*, followed by *Penicillium*, *Fusarium*, and *Alternaria* spp., a pattern consistent with surveys from other hazelnut-producing regions [24]. Comparable genera have also been reported in studies on defective hazelnuts, in which they presented a broad mycobiota including *Alternaria*, *Aspergillus*, *Botryosphaeria*, *Diaporthe*, *Fusarium*, *Penicillium*, and *Pestalotiopsis* as prevalent taxa, with *Rhizopus*, *Cladosporium*, *Trichoderma*, *Botrytis*, *Trichothecium* and *Mucor* occurring less frequently (<10%) [29]. In this research, the analysis specifically focused on mycotoxigenic genera, particularly *A. flavus*, given their established relevance for AF contamination and food safety. Other studies corroborate this pattern, reporting high recovery of *Aspergillus* spp., including *A. flavus* and *A. parasiticus*, from hazelnuts in Turkey, Croatia, Iran, Egypt, Saudi Arabia, and the USA [8,27,30,31,32].

Storage played a limited role. Neither storage temperature nor in-shell conservation significantly affected fungal loads or a_w_, which remained stable throughout the storage period. This indicates that once nuts are properly dried, storage alone is insufficient to drive major growth shifts, provided moisture remains low. Instead, environmental conditions linked to production year and orchard location were the dominant factors shaping fungal prevalence, pointing to the primary role of environmental conditions before and during harvest. This limited influence of storage conditions is consistent with previous studies showing that, once hazelnuts are properly dried to safe moisture (≤6%), storage temperature exerts minimal effects on fungal proliferation and AF formation. Studies from Turkey, Italy and Croatia show that correctly dried hazelnuts (MC ≤ 6%) remain stable for months under ambient conditions and that spoilage during storage arises mainly from insufficient drying rather than storage temperature [26,27,33,34]. However, it should be noted that the limited effect of storage conditions observed in this study is strictly linked to the fact that hazelnuts entered the storage phase at sufficiently low a_w_. Under conditions of ineffective or delayed drying, storage has been shown to contribute substantially to fungal growth and AF accumulation [16,25].

Regarding AF contamination, orchard samples consistently showed very low contamination, always within EU legal limits, whereas market samples displayed extreme heterogeneity, with a few in-shell lots stored for prolonged periods showing AFB_1_ levels >450 µg/kg. Such outliers likely originate from uncontrolled harvesting, delayed drying, or poor storage, highlighting how deficiencies in traceability and post-harvest handling remain the main drivers of severe contamination. This agrees with other reports showing wide variability in AF levels in commercial hazelnuts, ranging from <0.1 µg/kg to >100 µg/kg [9,28,30,35], and confirms that inadequate drying and soil contact before harvest are major contributors [7,27,32].

Although *A. flavus* and AFs represent the major safety concern in hazelnuts, other mycotoxigenic fungi such as *Penicillium* and *Fusarium* spp. may contribute additional risks through the production of ochratoxin A, patulin, and fumonisins [36]. Recent surveys on Azerbaijani hazelnuts revealed that, in addition to AFs, low but measurable fumonisin levels (0.12–0.30 mg/kg) in Azerbaijani hazelnuts, reinforcing the need for multi-mycotoxin monitoring, since AFs remain the only regulated contaminants in hazelnuts under EU law [22].

Recent FAO assessments on the Azerbaijani hazelnut sector provide a broader framework for evaluating the scalability and feasibility of improved post-harvest management strategies [2]. According to FAO, most smallholders rely on traditional sun drying, typically performed on bare ground or concrete surfaces, with limited turning and high susceptibility to re-wetting, which mirrors the BD conditions tested in this study. To address these limitations, FAO evaluated renewable-energy-based drying and storage systems, including village-scale solar-powered mechanical dryers and small solar cold-storage units for local markets. Their modeling suggests that a nationwide reduction in AF risk would require approximately 607 solar units, with an estimated investment between USD 11.9–16.8 million, depending on battery configuration, and with substantial potential reductions in greenhouse-gas emissions. These findings underscore that while mechanical drying is objectively superior, requiring only 18–33 h instead of up to 30 days under sun-drying conditions, its scalability depends heavily on future financial and infrastructural support to the region [2].

Additionally, several technological approaches, including irradiation, ultrasound, plasma, and chemical decontamination, have been explored to reduce AF contamination [36,37]. Among them, cold atmospheric plasma shows promise for inactivating *Aspergillus* spp. while preserving nut quality [38,39]. However, due to cost and scalability limitations, preventive measures such as efficient drying, proper storage, rigorous sorting, and continuous monitoring remain the most effective and economically feasible solutions.

In conclusion, this study demonstrates that drying efficiency is the primary determinant of fungal load and AF risk in Azerbaijani hazelnuts. While controlled post-harvest management can virtually eliminate AF hazards, inadequate drying and poor traceability continue to threaten both public health and export competitiveness. Future efforts should aim to harmonize and improve drying standards, support technological upgrades for smallholders, and strengthen traceability systems to ensure the long-term safety and marketability of Azerbaijani hazelnuts.

## 4. Materials and Methods

### 4.1. Study Design and Sampling Sites

The study was conducted over three consecutive production seasons (2022–2023, 2023–2024, and 2024–2025) in Azerbaijan. Six hazelnut orchards were selected and monitored throughout the study. Two orchards were in the Khachmaz region (Orchard #2 and #3), three in Zaqatala (Orchard #4, #5 and #6), and one in Qabala (Orchard #1). These orchards represent smallholder farming systems characterized by heterogeneous tree varieties and ages, cultivation practices, and management strategies. Most orchards followed organic or low-input management without synthetic fertilizers or pesticides. One orchard (Orchard #6; >20 years old) was managed using manure as fertilizer and occasional irrigation, whereas another (Orchard #3; 18 years old) applied conventional fertilization and disease control practices (ammonium phosphate, manure, urea, Bordeaux mixture) and submersion irrigation.

In addition to the six monitored orchards, eight commercial samples were collected from local markets in Azerbaijan, four from Khachmaz and four from Zaqatala. Hazelnuts from Khachmaz belonged to the cultivar Khachmaz, while those from Zaqatala and Qabala belonged to the cultivar Ata-baba.

### 4.2. Sample Collection and Drying Treatments

In this study, field samples were collected exclusively from the tree. For each orchard and market source, an initial composite sample of 200 kg of freshly harvested hazelnuts (with husk) was collected immediately after harvest. Each lot was divided into two equal subsamples (100 kg each) and subjected to two distinct drying protocols:

(I) Good drying (GD): Drying was performed in a ventilated area with exposure to sunlight. Nuts were spread in a single uniform layer and mixed once per day to ensure homogeneous moisture removal (Supplementary Figure S1). After 3–4 days, when the husk became dry and easily detachable, nuts were manually dehusked. In-shell drying then continued under the same conditions (single-layer spreading, sun-exposed but rain-protected area) until the kernels reached <0.70 a_w_ (with residual kernel moisture content typically below 6%).

(II) Bad Drying (BD). BD treatment was designed to simulate suboptimal drying conditions frequently observed in smallholder systems. Nuts were first dried in shaded areas, kept in piles during the first week without any mixing to maintain higher humidity (Supplementary Figure S1). During the second week, nuts were intentionally re-wetted, receiving three water sprayings per day, each consisting of 5 L of warm water (~35 °C). No mixing was performed. In the third week, watering stopped and nuts were spread more loosely (but not in a single layer) for 3–4 days to allow the husk to dry. Manual dehusking followed.

In the fourth week, in-shell nuts were dried in a shaded, cool, fresh area (maximum temperature 35 °C), again avoiding any mixing, until kernel a_w_ fell below 0.70 (moisture content < 6%).

### 4.3. Post-Drying Sampling and Storage Design

For the eight market-derived samples, an additional storage-related variable was tested: hazelnuts were stored either in-shell or as shelled kernels throughout the storage period.

After drying, hazelnuts were deshelled using a locally manufactured mechanical sheller (no commercial model available), with the exception of samples tested for the in-shell storage variable. Subsamples of 300 g for shelled nuts and 600 g for in-shell nuts were collected, placed in jute bags, and shipped to Italy for laboratory analyses. Transport lasted 1–2 days, during which hazelnuts were shipped under ambient conditions without vacuum sealing.

Upon arrival at the laboratory, samples were manually shelled (if not already in kernel form). Kernels were ground (Moulinex, Groupe SEB, Ecully, France), vacuum-sealed, and stored at 8–10 °C until analysis.

Remaining dried hazelnuts from each drying treatment were then divided into two equal portions and stored under two temperature regimes: cold storage (CS): 8–10 °C; room temperature storage (RTS): 18–22 °C.

In this study, the term “post-harvest phase” refers to the period starting immediately after drying and ending prior to industrial processing. Three standardized post-harvest phases were defined: PH1 (November, immediately after drying and shipment), PH2 (February, ~3 months of storage), and PH3 (May, ~6 months of storage). Each phase therefore represents a specific storage duration under controlled temperature conditions. At each time point, a 300 g subsample of in-shell hazelnuts was collected and processed as described above.

The overall design included 20 samples at PH1: 6 orchard-derived samples × 2 drying treatments (GD + BD) = 12 samples; 8 market samples (4 GD + 4 BD). For PH2 and PH3, the same 20 lots were analyzed for both the two storage treatments, resulting in 40 samples per time point (Supplementary Tables S2 and S3).

The effect of temperature was assessed by comparing CS and RTS samples within each post-harvest phase (PH1, PH2, PH3). The effect of storage duration was assessed by comparing PH1, PH2, and PH3 within each temperature condition. This structure allows temperature-dependent and time-dependent effects to be evaluated independently.

### 4.4. Fungi Quantification and Colonies Identification

Grounded hazelnut samples were used to determine fungi colony-forming units per gram of hazelnut flour (CFU/g). To determine colony-forming units, 1 g of sample per orchard was diluted in 9 mL of peptone water 0.1%. Once homogenized (Vortex ZX3, Genelab S.r.l., Perugia, Italy), spread plate serial dilutions (10^−1^ to 10^−3^) were made, with five replicates each, adding 500 µg of suspension on Dichloran Rose Bengal Chloramphenicol Agar (DRBC Agar Base [Biolife Italiana S.r.l, Monza, Italy], 0.05 g chloramphenicol, 1 L water). Plates were incubated in the dark at 31 °C for 3 days.

After incubation, colonies were assigned to one of the target groups based on macro- and microscopic features: *Alternaria* spp., *A.* section *Flavi*, *A.* section *Nigri*, *Fusarium* spp. and *Penicillium* spp. [40,41,42,43,44]. Preliminary identification was carried out directly on DRBC agar plates by stereomicroscopic observation of colony morphology, including colony color, texture, growth pattern, margin characteristics and pigmentation of the reverse. *Alternaria* spp. were identified by their darkly pigmented, velvety colonies with irregular margins; *Penicillium* spp. by their fast-growing, blue-green to grey-green colonies with a powdery texture; *Fusarium* spp. by their cottony to floccose colonies with white to pink pigmentation; *A*. section *Nigri* by black, granular colonies; and *A.* section *Flavi* by yellow-green to olive-green colonies. Colonies belonging to each group were counted separately on every plate. For each dilution, the mean colony number was calculated across the five replicate plates, and values from plates within the countable range were used to estimate fungal load. Counts were then converted to CFU/g according to the formula: CFU/g = (mean colony count × dilution factor × 2), where factor 2 accounts for plating 0.5 mL of the initial 1:10 homogenate.

Suspected *A.* section *Flavi* colonies were further subcultured onto 5/2 agar plates (5% V-8 vegetable juice, 2% agar, pH 5.2) and incubated for 5–7 days at 31 °C for more detailed morphological examination. Identification was performed under a stereomicroscope based on macroscopic colony characteristics, including colony color, texture, margin, reverse appearance, and presence or absence of sclerotia [23,40,45]. Typical *A. flavus*-like colonies displayed an ivy-green coloration with a granular to floccose texture, while *A. parasiticus*-like colonies appeared cress-green and more velvety.

Microscopic confirmation at the genus level was conducted by preparing wet mounts in sterile water to observe conidiophore and conidia structures. The presence of unbranched conidiophores terminating in globose vesicles bearing chains of rough-walled conidia was consistent with *Aspergillus* morphology. No micrometric measurements or species-level distinctions were made at this stage; molecular assays were subsequently used to confirm species identity.

### 4.5. Molecular Confirmation of A. Section Flavi Isolates

A total of 25 isolates, randomly selected among those morphologically identified as *A.* section *Flavi*, were subjected to species confirmation using SYBR Green quantitative PCR (qPCR). Isolates were firstly purified by single-spore isolations [41]. Monosporic cultures were inoculated into Potato Dextrose Broth (PDB) (for 1 L of PDB, 400 mL of potato-based extract, 20 gr of glucose, sterile water to volume) and incubated on an orbital shaker at 31 °C for 2 days to promote biomass accumulation.

Mycelial pellets were then harvested by centrifugation, and genomic DNA was extracted using the commercial extraction kit Nucleo Spin Plant II (Macherey-Nagel, Düren, Germany) following manufacturer guidelines. The obtained DNA was evaluated for purity and quantity by NanoDrop 2.0 spectrophotometer (ThermoFisher Scientific, Wilmington, DE, USA).

Isolates were identified through SYBR Green real-time PCR [46]. Reactions were performed in a total reaction volume of 20 µL (10 µL of GoTaq qPCR 2X MM [Promega, Madison, WI, USA, item A6101], 0.6 µL each of the primers for *A. flavus* [FLAVIQ1-5′ GTC GTC CCC TCT CCG G 3′, FLAQ2-5′ CTGGAAAAAGATTGATTTGCG 3′] and *A. parasiticus* [FLAVIQ1-5′ GTC GTC CCC TCT CCG G 3′, PARQ2-5′ GAA AAA ATG GTT GTT TTG CG 3′] [Eurofins Scientific, Torino, Italy], 0.4 µL of MgCl_2_ [Promega, Madison, WI, USA, item A6101], 0.16 µL of CXR [Promega, Madison, WI, USA, item A6101], 5 µL of DNA, and ultrapure water to volume).

Amplification was performed in a StepOnePlus Real-Time PCR System (ThermoFisher Scientific, Wilmington, DE, USA) with the following cycling conditions: 10 min at 95 °C, 40 cycles of 15 s at 95 °C, 1 min at 60 °C, with melting curve analysis performed from 60 to 95 °C, using a 0.3 °C/30 s increment.

Each run included positive controls (*A. flavus*: ITEM 8069; *A. parasiticus*: ITEM 18299) and negative controls (nuclease-free water). Cross-reactivity was checked by including *A. parasiticus* DNA when amplifying with *A. flavus* primers and vice versa. Isolates were assigned to species based on melting temperatures (*A. flavus* Tm: 88.5 °C; *A. parasiticus* Tm: 89.7 °C) and melting curves consistent with reference strains.

### 4.6. Aflatoxin Analysis

AF analysis was carried out according to UNI EN ISO 16050:2011 protocol [47]. For each sample, 50 g of hazelnut flour were mixed with 10 gr of sodium chloride, 150 mL of methanol and 100 mL of sterile water for 1 min. Samples were filtered with paper filters (Falc Instruments, Treviglio, Italy) and diluted with and equal quantity of sterile water (5 + 5, *v*/*v*). 10 mL of each diluted extract was injected at a flow rate of 2 mL min^−1^ in Easi Extract Aflatoxin immunoaffinity columns (R-Biopharm Rhone Ltd., Glasgow, Scotland, UK), containing a gel suspension of monoclonal antibody specific for the detection of AFB_1_, AFB_2_, AFG_1_ and AFG_2_.

After washing the column twice with 10 mL of sterile water and drying it with air flow, AFs were eluted with 1 mL of methanol, washed with 1 mL of deionised water and finally collected into 2 mL glass vials. When necessary, sterile water was used to bring glass vials to a total volume of 2 mL. The solution was analysed by reversed phase HPLC (Thermo Scientific Vanquish Core HPLC Systems, Thermo Scientific, Waltham, MA, USA) using post-column derivation and fluorescence detection. The column was a Supelcosil LC-18 (Merck KGaA, Darmstadt, Germany) and the mobile phase was H_2_O-CH_3_CN-CH_3_OH (3 + 1 + 1, *v*/*v*/*v*) at a flow rate of 1 mL min^−1^. AF production was quantified in µg kg^−1^ and the limit of detection was 0.2 µg kg^−1^.

### 4.7. Water Activity Measurement

a_w_ was determined on hazelnut samples from the 2024/2025 production season. Measurements were performed monthly, starting in October 2024 and continuing until June 2025, covering all experimental groups and storage conditions. a_w_ measurements were performed with AquaLab Pawkit (Graintec Scientific, Toowoomba, QLD, Australia), employing 3 g of hazelnut kernel, with three replicates for each measurement.

### 4.8. Statistical Analysis

Statistical analyses were performed with IBM SPSS Statistics software, version 30.0 (Chicago, IL, USA). Data on *A.* section *Flavi*, *Alternaria* spp., *A.* section *Nigri*, *Fusarium* spp. and *Penicillium* spp. CFU/g of hazelnuts were log_10_ transformed before statistical analysis and subjected to analysis of variance (ANOVA). In examining samples from selected orchards, each factor—region, year, post-harvest phase, drying technique, and storage temperature—was individually assessed first. Then, combinations of only the significant factors were considered. For market samples, an extra factor, storage in shell/shelled, was included. Tukey’s test was employed to compare means and identify significant differences.

## Figures and Tables

**Table 1:** Combined analysis of variance (ANOVA) of log_10_ (CFU/g) values for *Aspergillus* section *Flavi*, *Aspergillus* section *Nigri*, *Alternaria* spp., *Fusarium* spp., and *Penicillium* spp., obtained from hazelnuts collected in six selected orchards in Azerbaijan across three production years.

log_10_ (CFU/g)—Selected Orchards										
Factors	*A.* Section *Flavi*		*A.* Section *Nigri*		*Alternaria* spp.		*Fusarium* spp.		*Penicillium* spp.	
Orchard (O)	**		**		n.s		n.s		n.s	
1	0.8	^b^	1.3	^bc^	1.0		1.9		1.8	
2	1.5	^a^	2.1	^a^	1.1		1.4		1.5	
3	1.2	^ab^	1.6	^ab^	1.0		1.8		2.1	
4	0.8	^b^	0.9	^c^	1.2		1.8		1.6	
5	1.1	^ab^	1.1	^c^	1.0		2.1		1.6	
6	0.9	^b^	0.9	^c^	0.8		1.6		1.9	
Year (Y)	**		**		**		**		**	
2022	0.7	^b^	0.9	^b^	1.2	^a^	1.2	^b^	1.4	^b^
2023	1.7	^a^	1.9	^a^	1.6	^a^	2.9	^a^	2.1	^a^
2024	0.7	^b^	1.2	^b^	0.3	^b^	1.2	^b^	1.8	^ab^
Drying (D)	**		*		n.s		**		**	
GD	0.8	^b^	1.2	^b^	1.1		1.5	^b^	1.4	^b^
BD	1.3	^a^	1.5	^a^	1.0		2.0	^a^	2.1	^a^
Interactions										
O × Y	**		*		*		n.s		n.s	
O × D	n.s		*		n.s		n.s		n.s	
Y × D	**		n.s		n.s		n.s		n.s	
O × Y × D	n.s		n.s		n.s		n.s		n.s	

Notes: ** *p* < 0.01; * *p* < 0.05; n.s for not significant. Different letters indicate significant differences according to Tukey’s test.

**Table 2:** Combined analysis of variance (ANOVA) of log_10_ (CFU/g) values for *Aspergillus* section *Flavi*, *Aspergillus* section *Nigri*, *Alternaria* spp., *Fusarium* spp., and *Penicillium* spp., obtained from hazelnuts collected from Azerbaijani market.

log_10_ (CFU/g)—Market Samples										
Factors	*A.* Section *Flavi*		*A.* Section *Nigri*		*Alternaria* spp.		*Fusarium* spp.		*Penicillium* spp.	
Market Source (M)	**		**		*		n.s		**	
1	1.5	^a^	1.3	^ab^	1.0	^ab^	1.6		2.4	^bc^
2	0.6	^b^	1.2	^b^	1.0	^ab^	1.7		1.8	^c^
3	1.3	^ab^	2.1	^a^	0.9	^ab^	1.9		3.3	^ab^
4	1.4	^ab^	1.5	^ab^	0.9	^ab^	2.0		1.8	^c^
5	0.6	^b^	1.3	^ab^	1.0	^ab^	1.9		2.7	^abc^
6	1.6	^a^	1.8	^ab^	1.5	^a^	1.1		3.7	^a^
7	0.9	^ab^	1.0	^b^	1.4	^a^	2.5		2.2	^c^
8	0.7	^b^	1.2	^ab^	0.3		1.7		2.2	^bc^
Year (Y)	**		**		**		**		**	
2022	1.0	^b^	1.3	^b^	0.8	^b^	1.1	^c^	2.4	^b^
2023	1.5	^a^	1.8	^a^	1.4	^a^	2.6	^a^	3.0	^a^
2024	0.7	^b^	1.2	^b^	0.7	^b^	1.8	^b^	2.2	^b^
Interactions										
M × Y	**		**		n.s		*		**	

Notes: ** *p* < 0.01; * *p* < 0.05; n.s for not significant. Different letters indicate significant differences according to Tukey’s test.

**Table 3:** Aflatoxin B_1_ (AFB_1_, μg/kg) and total aflatoxins (AFs tot, μg/kg) detected in hazelnut samples collected from selected orchards and market sources, after 6-month storage (PH3). Samples differ by drying method (good drying-GD, bad drying-BD), storage temperature (cold storage-CS, room temperature storage-RTS), storage condition (in-shell vs. shelled) and production year (2022–2023).

Sample Source	Drying	Storage T	Storage in Shell	Year	AFB_1_ (μg/kg)	AFB_2_ (μg/kg)	AFG_1_ (μg/kg)	AFG_2_ (μg/kg)	AF tot (μg/kg)
Market 6	BD	RTS	yes	2022	<0.2	<0.2	<0.2	0.48	0.48
Market 7	GD	RTS	yes	2022	161.33	6.18	45.49	1.42	214.42
Market 8	GD	CS	yes	2022	2.32	<0.2	<0.2	<0.2	2.65
Market 8	GD	RTS	yes	2022	0.24	<0.2	<0.2	<0.2	0.24
Market 5	BD	CS	yes	2023	12.06	0.87	<0.2	<0.2	12.93
Market 6	BD	CS	yes	2023	454.54	65.56	<0.2	<0.2	520.1
Orchard 1	BD	RTS	no	2023	0.28	<0.2	<0.2	<0.2	0.28
Orchard 2	BD	RTS	no	2023	1.46	<0.2	<0.2	<0.2	1.46

Notes: orchard and market samples not included in the table yielded AF levels < LOD (<0.2 µg/kg).

## Data Availability

The original contributions presented in the study are included in the article and in its Supplementary Materials, further inquiries can be directed to the corresponding author.

## Supplementary Materials

The following supporting information can be downloaded at https://www.mdpi.com/article/10.3390/toxins18010038/s1. Table S1: Monthly water activity (a_w_) values of hazelnut samples collected from selected orchards and market sources during the 2024/2025 production season; Table S2: List of all hazelnut samples collected from the selected orchards and tested throughout the three years of the study (2022–2024); Table S3: List of hazelnut samples recovered from the Azerbaijani market throughout three years (2022–2024). Figure S1: Description of the drying procedures applied in the study.

## Abbreviations

The following abbreviations are used in this manuscript:

AFs	Aflatoxins
AFB_1_	Aflatoxin B1
AFB_2_	Aflatoxin B2
AFG_1_	Aflatoxin G1
AFG_2_	Aflatoxin G2
RASFF	Rapid Alert System for Food and Feed
CFU	Colonies forming units
GD	Good drying
BD	Bad drying
CS	Cold storage
Cold storage	Room temperature storage
ANOVA	Analysis of variance
a_W_	Water activity
PH1	Post-harvest phase 1 (November)
PH2	Post-harvest phase 2 (February)
PH3	Post-harvest phase 3 (May)
°C	Celsius degrees
EU	Europe
PCR	Polymerase chain reaction
DNA	Deoxyribonucleic acid
ITEM	Agri-Food Toxigenic Fungi Culture Collection (CNR-ISPA, Bari)
HPLC	High-performance liquid chromatography
H_2_O	Dihydrogen oxide/water
CH_3_CN	Acetonitrile
CH_3_OH	Methanol
MC	Moisture content
