# Supplementary material for: Effect of Post-Harvest Management on Aspergillus flavus Growth and Aflatoxin Contamination of Stored Hazelnuts
Source: Toxins (Basel). 2026 Jan 11;18(1):38. doi: 10.3390/toxins18010038 (PMC12846331; doi:10.3390/toxins18010038)
Supplement: Supplementary file 1 [file toxins-18-00038-s001.zip › toxins-4067593-supplementary.pdf]

Supplementary Materials: Effect of Post-Harvest Management on *Aspergillus flavus* Growth and Aflatoxin Contamination of Stored Hazelnuts

Alessia Casu, Giorgio Chiusa, Eugenio Zagottis, Giuseppe Genova and Paola Battilani

Supplementary Table S1. Monthly water activity (a_w) values of hazelnut samples collected from selected orchards and market sources during the 2024/2025 production season. Samples were characterized by drying method (GD, BD), storage temperature (CS, RTS), storage condition (in-shell vs shelled), and geographical origin. Measurements were performed from October 2024 to June 2025.

Sample Code	Drying method	Storage Temperature	Storage in shell	Geographical Origin	Aw October	Aw November	Aw December	Aw January	Aw February	Aw March	Aw April	Aw May	Aw June
Kagr n10	BD	CS	no	Khachmaz	0.64	0.62	0.61	0.61	0.61	0.62	0.63	0.64	0.63
K29 n6	BD	CS	no	Khachmaz	0.62	0.61	0.61	0.61	0.61	0.62	0.64	0.64	0.63
Z27 n26	BD	CS	no	Zaqatala	0.64	0.67	0.66	0.62	0.62	0.62	0.64	0.63	0.65
Z41 n38	BD	CS	no	Zaqatala	0.63	0.63	0.63	0.63	0.63	0.64	0.65	0.64	0.65
ZRC n39	BD	CS	no	Zaqatala	0.63	0.66	0.64	0.64	0.63	0.63	0.64	0.64	0.62
G23 n16	BD	CS	no	Qabala	0.67	0.66	0.65	0.65	0.63	0.62	0.63	0.64	0.65
Kagr n10	BD	RTS	no	Khachmaz	0.64	0.64	0.64	0.64	0.63	0.63	0.64	0.63	0.66
K29 n6	BD	RTS	no	Khachmaz	0.62	0.64	0.62	0.62	0.62	0.61	0.63	0.63	0.64
Z27 n26	BD	RTS	no	Zaqatala	0.64	0.65	0.65	0.64	0.62	0.63	0.63	0.63	0.66
Z41 n38	BD	RTS	no	Zaqatala	0.63	0.64	0.65	0.63	0.62	0.62	0.63	0.63	0.66
ZRC n39	BD	RTS	no	Zaqatala	0.63	0.67	0.64	0.64	0.64	0.64	0.64	0.63	0.64
G23 n16	BD	RTS	no	Qabala	0.67	0.67	0.66	0.65	0.65	0.63	0.62	0.63	0.65
Kagr n10	GD	CS	no	Khachmaz	0.64	0.62	0.62	0.62	0.61	0.62	0.63	0.64	0.64
K29 n6	GD	CS	no	Khachmaz	0.62	0.61	0.61	0.62	0.62	0.63	0.64	0.64	0.64
Z27 n26	GD	CS	no	Zaqatala	0.63	0.67	0.65	0.61	0.61	0.62	0.63	0.64	0.64

Z41 n38	GD	CS	no	Zaqatala	0.62	0.65	0.65	0.65	0.63	0.64	0.65	0.65	0.65
ZRC n39	GD	CS	no	Zaqatala	0.63	0.65	0.65	0.65	0.64	0.64	0.65	0.64	0.65
G23 n16	GD	CS	no	Qabala	0.67	0.66	0.64	0.64	0.61	0.62	0.64	0.64	0.65
Kagr n10	GD	RTS	no	Khachmaz	0.64	0.64	0.63	0.63	0.63	0.63	0.64	0.64	0.66
K29 n6	GD	RTS	no	Khachmaz	0.62	0.64	0.63	0.64	0.64	0.64	0.64	0.63	0.65
Z27 n26	GD	RTS	no	Zaqatala	0.63	0.67	0.67	0.67	0.64	0.65	0.66	0.64	0.65
Z41 n38	GD	RTS	no	Zaqatala	0.62	0.63	0.66	0.65	0.63	0.65	0.65	0.63	0.66
ZRC n39	GD	RTS	no	Zaqatala	0.63	0.65	0.64	0.64	0.63	0.64	0.64	0.63	0.66
G23 n16	GD	RTS	no	Qabala	0.67	0.66	0.66	0.65	0.63	0.64	0.65	0.64	0.65
Zs9KB	BD	CS	no	Zaqatala	0.74	0.67	0.66	0.64	0.63	0.63	0.64	0.63	0.63
Ks11SB	BD	CS	yes	Khachmaz	0.73	0.63	0.63	0.61	0.62	0.63	0.61	0.6	0.61
Zs10SB	BD	CS	yes	Zaqatala	0.72	0.67	0.66	0.66	0.64	0.64	0.63	0.63	0.62
Ks12KB	BD	CS	no	Khachmaz	0.74	0.68	0.65	0.63	0.62	0.63	0.64	0.64	0.63
Zs9KB	BD	RTS	no	Zaqatala	0.74	0.64	0.62	0.62	0.62	0.63	0.64	0.64	0.66
Ks11SB	BD	RTS	yes	Khachmaz	0.73	0.63	0.63	0.61	0.60	0.62	0.63	0.61	0.63
Zs10SB	BD	RTS	yes	Zaqatala	0.72	0.62	0.62	0.62	0.60	0.60	0.61	0.62	0.66
Ks12KB	BD	RTS	no	Khachmaz	0.74	0.65	0.65	0.65	0.64	0.63	0.64	0.64	0.65
KagrKG	GD	CS	no	Khachmaz	0.72	0.70	0.70	0.65	0.64	0.64	0.64	0.63	0.64
ZagrKG	GD	CS	no	Zaqatala	0.73	0.71	0.71	0.67	0.67	0.65	0.65	0.64	0.63
KagrSG	GD	CS	yes	Khachmaz	0.71	0.72	0.70	0.66	0.64	0.64	0.65	0.64	0.63
ZagrSG	GD	CS	yes	Zaqatala	0.79	0.71	0.70	0.66	0.64	0.63	0.64	0.63	0.63
KagrKG	GD	RTS	no	Khachmaz	0.72	0.69	0.69	0.68	0.67	0.65	0.65	0.64	0.65
ZagrKG	GD	RTS	no	Zaqatala	0.73	0.74	0.72	0.66	0.65	0.66	0.66	0.65	0.65
KagrSG	GD	RTS	no	Khachmaz	0.71	0.70	0.67	0.65	0.64	0.64	0.63	0.62	0.67
ZagrSG	GD	RTS	yes	Zaqatala	0.79	0.69	0.63	0.56	0.56	0.54	0.58	0.59	0.66

Supplementary Table S2. List of all hazelnut samples collected from the selected orchards and tested throughout the three years of the study (2022-2024). Each sample code represents a different orchard located in Azerbaijan (Khachmaz, Zaqatala and Qabala regions). Drying method, storage temperature and sample weight are indicated for each sample.

Orchard[1]	Sample Code[1]	Drying Method[2]	Storage Temperature[3]	Geographical Origin	Sample Weight (g)
1	G21 n18	BD	CS	Qabala	300
2	K29 n6	BD	CS	Khachmaz	300
3	Kagr n10	BD	CS	Khachmaz	300
4	Z27 n26	BD	CS	Zaqatala	300
5	Z41 n38	BD	CS	Zaqatala	300
6	ZRC n39	BD	CS	Zaqatala	300
1	G21 n18	GD	CS	Qabala	300
2	K29 n6	GD	CS	Khachmaz	300
3	Kagr n10	GD	CS	Khachmaz	300
4	Z27 n26	GD	CS	Zaqatala	300
5	Z41 n38	GD	CS	Zaqatala	300
6	ZRC n39	GD	CS	Zaqatala	300
1	G21 n18	BD	RTS	Qabala	300
2	K29 n6	BD	RTS	Khachmaz	300
3	Kagr n10	BD	RTS	Khachmaz	300
4	Z27 n26	BD	RTS	Zaqatala	300
5	Z41 n38	BD	RTS	Zaqatala	300
6	ZRC n39	BD	RTS	Zaqatala	300
1	G21 n18	GD	RTS	Qabala	300
2	K29 n6	GD	RTS	Khachmaz	300
3	Kagr n10	GD	RTS	Khachmaz	300
4	Z27 n26	GD	RTS	Zaqatala	300
5	Z41 n38	GD	RTS	Zaqatala	300
6	ZRC n39	GD	RTS	Zaqatala	300

Notes:

[1] Each sample code represents a different orchard located either in Khachmaz, Zaqatala e Qabala regions of Azerbaijan (K= Khachmaz, Z= Zaqatala, G= Qabala). Orchards are identified with codes ranging from #1 to #6.

[2] BD stands for Bad Drying, while GD indicates Good Drying.

[3] CS is indicated for samples stored at cold temperatures (8-10°C), while RTS represents samples kept at room temperature (18-22°C).

Supplementary Table S3. List of hazelnut samples recovered from the Azerbaijani market throughout three years (2022-2024). For each sample, it is indicated whether the storage was conducted in shell or in kernel, together with the drying conditions and storage temperature tested. Geographical origin and sample weight at delivery are also reported for each sample.

Market Source[1]	Sample Code[1]	Drying Method[2]	Storage Temperature[3]	Storage in Shell	Geographical Origin	Sample Weight (g)
#1	Zs9KB	BD	CS	no	Zaqatala	600
#2	KagrKG	GD	CS	no	Khachmaz	600
#3	Ks12KB	BD	CS	no	Khachmaz	600
#4	ZagrKG	GD	CS	no	Zaqatala	600
#5	Zs10SB	BD	CS	yes	Zaqatala	600
#6	Ks11SB	BD	CS	yes	Khachmaz	600
#7	ZagrSG	GD	CS	yes	Zaqatala	600
#8	KagrSG	GD	CS	yes	Khachmaz	600
#1	Zs9KB	BD	RTS	no	Zaqatala	600
#2	KagrKG	GD	RTS	no	Khachmaz	600
#3	Ks12KB	BD	RTS	no	Khachmaz	600
#4	ZagrKG	GD	RTS	no	Zaqatala	600
#5	Zs10SB	BD	RTS	yes	Zaqatala	600
#6	Ks11SB	BD	RTS	yes	Khachmaz	600
#7	ZagrSG	GD	RTS	yes	Zaqatala	600
#8	KagrSG	GD	RTS	yes	Khachmaz	600

Notes:

[1] Each sample was collected from the Azerbaijani market. For each of them, only the general regional provenience is known (Khachmaz or Zaqatala). Market sources are identified with codes ranging from #1 to #8.

[2] Drying method is reported either as GD (good drying) or BD (bad drying).

[3]Hazelnut samples were subjected to two different temperature regimes: CS (cold storage, 8-10°C) and RTS (room temperature storage, 18-22°C).

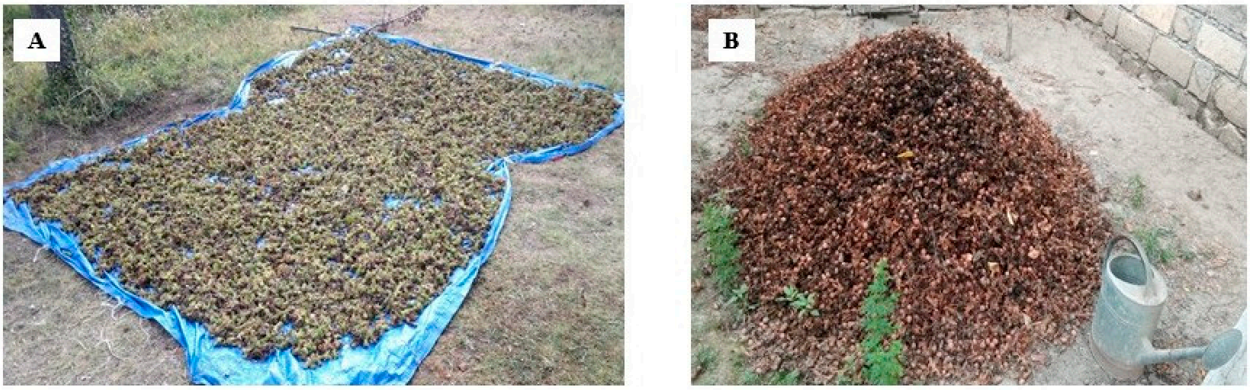

Supplementary Figure S1. Description of the drying procedures applied in the study. (**A)** Good drying (GD) procedure, with hazelnuts spread in a single, thin layer and regularly turned under a ventilated, sun-exposed area. (**B)** Bad drying (BD) procedure, with hazelnuts kept in piled heaps in a shaded, poorly ventilated area and periodically re-wetted.
